# Validity of the Montreal Cognitive Assessment–Basic (MoCA-B) in a Dutch Memory Clinic

**DOI:** 10.1097/WAD.0000000000000729

**Published:** 2026-05-28

**Authors:** Roy P.C. Kessels, Floor S. van Bergen, Calijn M.A. Vogel, Marloes Smarius, Iris S. Pouwels, Paul D. Dautzenberg

**Affiliations:** * Radboud University, Donders Institute for Brain, Cognition and Behaviour; ‡ Radboud University Medical Center, Radboudumc Alzheimer Center; ¶ iPractice, Nijmegen; † Vincent van Gogh Institute for Psychiatry, Venray; § Department of Medical Psychology, Jeroen Bosch Hospital; ** Brain Research Center, ’s-Hertogenbosch; ∥ Syndion, Gorinchem; # GGZ Westelijk Noord Brabant, Halsteren, The Netherlands

**Keywords:** cognitive screen, low literacy, education, psychometric analysis, cognitive testing, memory clinic

## Abstract

People with low educational levels or low literacy skills tend to obtain lower scores on cognitive screens, even in the absence of cognitive impairments, possibly resulting in false positive diagnoses. The Montreal Cognitive Assessment–Basic (MoCA-B) has been developed to overcome this, but studies so far are limited to low-income countries. This study examined the MoCA-B [total score and Memory Index Score (MIS)] in a European memory clinic context. Fifty-five controls, 37 patients with mild cognitive impairment and 47 dementia patients were included. Results showed that a MoCA-B total cutoff score <25 is valid for distinguishing controls from MCI or dementia patients. The MoCA-B MIS showed an acceptable diagnostic accuracy for controls versus MCI (<12) and controls versus dementia (<11). No valid cutoff score could be established for MCI versus dementia patients. Our findings show that the MoCA-B can also be validly applied in a European memory clinic setting.

The prevalence of subjective cognitive decline (SCD), mild cognitive impairment (MCI), and all-cause dementia in older people varies greatly based on epidemiological studies, ranging from 12.9% to 65.3% for SCD, 17.2% to 31.3% for MCI, and 4.5% to 22.7% for dementia.^1,2^ Low education is a risk factor for developing late-life MCI or dementia,^3^ while a low education or illiteracy also bears the risk of false positive diagnoses and is associated with lower performances on cognitive screens.^3,4^ However, cognitive screens do not always take educational attainment or literacy into account.^5^

The Montreal Cognitive Assessment–Basic (MoCA-B), a modified version of the MoCA, was developed to overcome this limitation, facilitating its use in individuals with a low education level or low literacy by eliminating literacy-dependent items. Items heavily influenced by education were substituted by literacy-independent items that measure the same cognitive function.^6^ To date, the MoCA-B has only been validated for detecting MCI or dementia in Thailand,^6^ Vietnam,^7^ China,^8^ Egypt,^9^ Iran,^10^ and Ecuador.^11^ However, the number of people with low literacy in Western, high-income regions is also substantial, with estimates of 80 million people having poor literacy skills in Europe, for instance.^12^ This stresses the need for validated cognitive screens for use in low-literacy individuals. This study validates the MoCA-B for distinguishing between cognitively unimpaired older adults and people with MCI or dementia in a memory clinic in the Netherlands.

## METHODS

### Participants

Participants with MCI and dementia were recruited from the Jeroen Bosch Hospital, ‘s-Hertogenbosch, the Netherlands. Healthy controls were recruited from their relatives or spouses. Clinical diagnoses were made in a multidisciplinary way, based on the clinical interview, neuropsychological examination, physical examination, neurological assessment, and/or neuroimaging findings, in accordance with the diagnostic criteria for MCI and all-cause dementia. All participants were aged 50 or older and fluent in Dutch. Participants were excluded if they had impairments in vision or hearing, current psychiatric disorders, alcohol abuse or substance use, other brain disorders, experienced delirium <3 months before inclusion, or used psychoactive medication affecting cognition. Education level was recorded as 7 categories (1=less than primary school; 7=university degree) and categorized as low, average or highly educated based on the Dutch educational system.^13^

### Materials

The authorized Dutch version of the MoCA-B was used (www.mocacognition.com). The MoCA-B consists of 9 items and covers 6 cognitive domains: executive functioning, episodic memory, language, orientation, visuoperception, and attention. The total scores ranges from 0 to 30 and was not corrected for education. We expanded the administration procedure with the Memory Index Score (MIS) that is part of the conventional MoCA,^14^ in which the memory items are not only tested using free recall, but also through cued recall and multiple-choice recognition, resulting in a separate scoring ranging from 0 to 15.

### Statistical Analyses

Analyses were performed using IBM SPSS 29.0. Descriptive variables, the MoCA-B item scores, total score and MIS were compared across groups using χ^2^ for dichotomous variables, Kruskal-Wallis *H* for ordinal scales and analysis of variance for continuous variables, followed by the appropriate post hoc tests. Spearman ρ correlations and 95% CI were computed between education level (1 to 7) and the MoCA-B total score and MIS. Receiver operating characteristics (ROC) analyses were performed comparing controls versus MCI, controls versus dementia and MCI versus dementia. Areas under the curve (AUC) with 95% CI were computed and interpreted as having excellent (>0.9), moderate (0.7 to 0.9) or poor (0.5 to 0.7) diagnostic accuracy. Sensitivity and specificity were computed for specific cutoff points, and the optimal cutoff point was determined using Youden *J*, where a sensitivity and specificity >0.8 can be considered acceptable and >0.9 excellent. Separate ROC analyses were also computed for the 3 educational groups, and their AUCs were compared statistically using MedCalc (www.medcalc.org).

## RESULTS

Fifty-five control participants, 37 MCI patients and 47 individuals with dementia were included. Table 1 shows the demographic and diagnostic variables and the MoCA-B performance. The controls were slightly younger than the 2 patient groups and included more women. MCI patients performed worse than the controls on the MoCA-B total score and MIS but better than the dementia group. On the MoCA individual items, MCI patients performed worse than controls on all but the naming item. Dementia patients performed worse than controls on all items, but only worse than the MCI patients on executive function, fluency, orientation, calculation, delayed recall, and naming. Education level (1 to 7) was neither statistically significantly associated with the MoCA-B total score (ρ=0.109, 95% CI: −0.063 to 0.275, *P*=0.201) nor with the MoCA-B MIS (ρ=0.010, 95% CI: −0.163 to 0.182, *P*=0.906).

**TABLE 1:** Demographic and Diagnostic Characteristics and Results on the MoCA-B for the Healthy Controls, the MCI Group and the People With Dementia

	Mean (SD) or n				
	CON (n=55)	MCI (n=37)	Dementia (n=47)	Overall statistic	Overall *P*
Sex (m/f)	8/47	26/11***	29/18***	χ^2^(2)=35.4	<0.001
Age	69.4 (9.3)	75.1 (6.0)***	76.2 (7.1)***	*F*(2,136)=11.0	<0.001
Education level				*H*(2)=0.7	0.706
Low	12	10	13		
Average	15	16	15		
High	28	11	19		
Education classification (1-7)‡	5 (2-7)	5 (1-7)	5 (4-7)	*H*(2)=0.3	0.859
Years of education	11.7 (3.1)	11.8 (3.2)	12.1 (3.2)	F(2136)=0.3	0.761
CDR (0.5/1/2)		37/0/0	0/44/3		
Etiology					
AD			20		
VaD			2		
Mixed dementia			13		
Lewy body disease			4		
PPA			4		
bvFTD			2		
Unknown			2		
MoCA-B					
Executive function (0/1)	15/40	12/25	29/18***,##	*H*(2)=13.7	<0.001
Fluency (0/1/2)	1/29/35	5/26/6***	20/24/3***,##	*H*(2)=51.2	<0.001
Orientation (0/1/2/3/4/5/6)	0/0/0/0/0/3/52	0/0/0/0/2/7/28**	0/0/0/1/7/15/24***,#	*H*(2)=26.2	<0.001
Calculation (0/1/2/3)	1/6/5/43	4/6/9/18**	5/15/17/10***,#	*H*(2)=28.6	<0.001
Abstraction (0/1/2/3)	0/3/9/43	1/5/12/19**	1/5/22/19***	*H*(2)=14.7	<0.001
Delayed recall (0/1/2/3/4/5)	0/1/7/11/20/16	12/9/4/8/3/1***	26/7/7/5/1/1***,#	*H*(2)=69.8	<0.001
Visuoperception (0/1/2/3)	0/0/4/51	0/0/9/28*	3/1/16/27***	*H*(2)=18.2	<0.001
Naming (0/1/2/3/4)	0/0/9/46	0/1/9/27	2/4/16/25***,#	*H*(2)=12.9	<0.001
Attention (0/1/2/3)	0/4/1/50	5/6/9/17***	10/11/9/17***	*H*(2)=34.6	<0.001
MoCA-B Total score	27.0 (2.2)	21.9 (3.0)***	18.7 (3.8)***,###	*F*(2136)=97.8	<0.001
MoCA-B MIS	13.2 (1.8)	8.8 (3.4)***	6.7 (3.9)***,##	*F*(2134)=55.9	<0.001

AD indicates Alzheimer disease; bvFTD, behavioral variant Frontotemporal dementia; CDR, clinical dementia rating; PPA, primary progressive aphasia; VaD, vascular dementia.

‡ mode (range).

Post hoc group comparisons:

* *P*<0.05.

** *P*<0.01.

*** *P*<0.001 [compared with controls].

# *P*<0.05.

## *P*<0.01.

### *P*<0.001 [compared with MCI].

**FIGURE 1:**
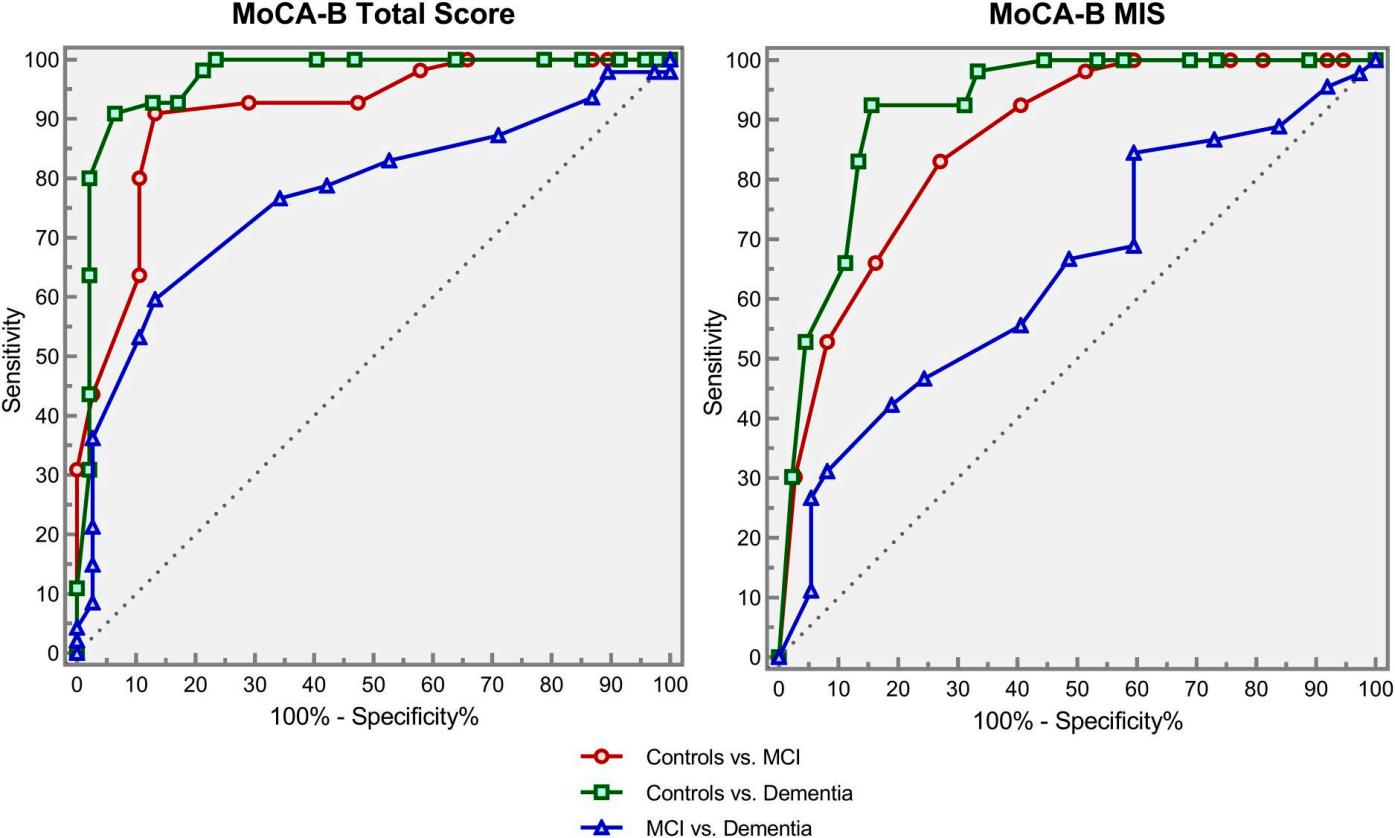
Receiver operating characteristic (ROC) curves for the MoCA-B total score (left) and the MoCA-B MIS (right) comparing the controls to the MCI and dementia group and comparing MCI to dementia patients.

Comparing the control group to the MCI group using ROC analyses (Fig. 1) resulted in excellent diagnostic accuracy for the MoCA-B total score for controls versus MCI (AUC: .946 [95% CI: 0.888-1.004], *P*<0.0005), and controls versus dementia (AUC: 0.977 [95% CI: 0.939-1.015], *P*<0.0005). Diagnostic accuracy for the MoCA-B total score for MCI versus dementia was moderate [AUC: 0.741 (95% CI: 0.663-0.848), *P*<0.0005]. For the MoCA-B MIS, the diagnostic accuracy was moderate-to-excellent for controls versus MCI (AUC: 0.863 [95% CI: 0.785-0.940], *P*<0.0005) and excellent for controls versus dementia (AUC: 0.922 [95% CI: 0.867-0.977], *P*<0.0005). Diagnostic accuracy of the MoCA-B MIS for MCI versus dementia was poor (AUCL 0.660 [95% CI: 0.543-0.776], *P*<0.007). Table 2 shows the individual cutoff scores with their sensitivity and specificity and Youden *J*. For the MoCA-B total score, a cutoff of <25 was optimal for controls versus MCI and controls versus dementia, with (almost) excellent sensitivity and specificity. A cutoff of <20 for the total score was the best for MCI versus dementia, albeit with a poor sensitivity (.596). For the MoCA-B MIS, the optimal cutoff score was <12 for controls versus MCI, with a low sensitivity and acceptable specificity. A MoCA-B MIS <11 could best distinguish controls from dementia patients, with an acceptable sensitivity and excellent specificity. A MoCA-B MIS <11 could also best distinguish MCI from dementia patients, with an acceptable sensitivity, but poor specificity. Comparing the AUCs for the separate ROC analyses per education group (low/average/high) did neither result in significant differences for the total score (*Z*<1.31, *P*>0.19) nor the MIS (*Z*<1.24, *P*>0.22).

**TABLE 2:** Sensitivity, Specificity, and the Youden Index for the Different Cutoff Scores for the MoCA-B Total Score and MIS

	Controls vs. MCI				Controls vs. dementia				MCI vs. dementia			
MoCA-B	Cutoff	Sensitivity	Specificity	Youden *J*	Cutoff	Sensitivity	Specificity	Youden *J*	Cutoff	Sensitivity	Specificity	Youden *J*
Total score	<23	0.541	0.925	0.465	<23	0.830	0.925	0.754	<18	0.362	0.973	0.335
	<24	0.730	0.925	0.654	<24	0.872	0.925	0.797	<19	0.532	0.892	0.424
	**<25**	**0.892**	**0.906**	**0.798**	**<25**	**0.936**	**0.906**	**0.842**	**<20**	**0.596**	**0.865**	**0.461**
	<26	0.892	0.792	0.684	<26	0.979	0.792	0.771	<21	0.766	0.649	0.415
	<27	0.892	0.623	0.515	<27	0.979	0.623	0.601	<22	0.787	0.568	0.355
MIS	<9	0.486	0.981	0.468	<9	0.681	0.981	0.662	<9	0.681	0.514	0.194
	<11	0.595	0.925	0.519	<10	0.702	0.925	0.627	<10	0.702	0.405	0.108
	**<12**	**0.730**	**0.830**	**0.560**	**<11**	**0.851**	**0.925**	**0.776**	**<11**	**0.851**	**0.405**	**0.256**
	<13	0.838	0.660	0.498	<12	0.872	0.830	0.703	<12	0.872	0.270	0.143
	<14	0.919	0.528	0.447	<13	0.894	0.660	0.554	<13	0.894	0.162	0.056

Bold indicates the optimal cutoff scores.

## DISCUSSION

This study validated the Dutch version of the MoCA-B in a sample of MCI and dementia patients in a memory clinic setting, comparing them with healthy older controls. The MoCA-B total score had a (near) excellent diagnostic accuracy for distinguishing controls from MCI or dementia patients using a cutoff of <25. This cutoff is comparable to the original MoCA-B study in a Thai sample,^6^ but higher than other culturally adapted MoCA-B versions.^8–10^ For the MoCA-B MIS, a cutoff of <12 was best in distinguishing controls from MCI patients and <11 for distinguishing controls from dementia patients. Research on the MoCA-B MIS is to our knowledge limited to one study performed with the Chinese version, reporting a lower cutoff score for controls versus MCI (<9).^8^ Education level was not correlated with the MoCA-B total score or MIS. This substantiates the claims that the MoCA-B is less susceptible to education effects compared with the full MoCA.^6^ No valid cutoff scores could be established for distinguishing MCI from dementia patients (lacking either sensitivity or specificity). This can be explained by the relatively early stage of the dementia patients in our sample, as all were recruited from a memory clinic and almost all had a CDR of 1, likely resulting in a large overlap in cognitive (dys)function with the MCI patients.

Our study has several limitations. Although care was taken to include individuals with low education levels, the Netherlands are a high-income country where even older people had access to at least primary education. Thus, our definition of low education is incomparable to the low education and illiteracy found in low-income countries, especially in older generations. This is also illustrated by the finding that none of the individuals in our sample had ≤4 years of education, for which a 1-point education correction would have been required.^6^ Future studies should extend and replicate our results in more participants with low education levels or low-literacy skills, also using a culturally more diverse sample. Moreover, the validity of the MoCA-B MIS needs to be studied in future research, as to date, there is little empirical evidence that this potentially informative measure can be used to either diagnose dementia stages or dementia subtypes.^14^ Finally, future studies should compare the diagnostic validity and other psychometric characteristics of the MoCA-B with other available cognitive screens that can be used in patients who are either illiterate or have a low education level, such as the Rowland Universal Dementia Assessment Scale (RUDAS) or the Brief Assessment of Impaired Cognition (BASIC).^15^

In conclusion, our results suggest that the Dutch MoCA-B total score is valid for distinguishing controls from MCI or dementia patients, and that the MoCA-B MIS, albeit promising, needs to be interpreted with caution. We argue that the MoCA-B can be an helpful (yet additional) tool in the cognitive assessment of individuals with low literacy skills or low education levels.
